# Lectures on the Processes of Repair and Reproduction after Injuries

**Published:** 1850-02

**Authors:** James Paget

**Affiliations:** Professor of Anatomy and Surgery to the College


### RECORD OF MEDICAL SCIENCE.

### ANATOMY AND PHYSIOLOGY.

*Lectures on the processes of Repair and Reproduction after Injuries. Delivered at the Royal College of Surgeons of England.* By JAMES PAGET, F. R. C. S., Professor of Anatomy and Surgery to the College.

### LECTURE II.

MR. PRESIDENT AND GENTLEMEN,—In the last lecture I endeavoured to establish the principle, that the power exercised in the repair of injuries was the same as that put forth in the developement of the germ from the lowest to the most perfect state in the full formed animal; and that this power was not determined by the fact of a higher or lower position in the scale of animal life, but that it bears an inverse proportion to the power expended in the developement of the germ. I omitted all reference, then, to the amount of reparative power in the highest vertebrata. In the warm-blooded animals the only parts capable of being reproduced may be divided into three classes.

1. Those in which each part is replaced by a repetition of the act which formed the first; among these are, the blood and cuticle. 2. Those of the lower organization or chemical composition—the gelatinous tissues and the osseous system. 3. Those accessories to the organic structures—the blood-vessels, nerves, lymphatics. These include all that can be reproduced. I shall now devote the time allotted to this lecture to the consideration of the materials necessary to the processes of repair; and I hope I shall not err in limiting myself to certain typical instances. I propose to limit myself to wounds and fractures, and now to speak especially of the materials necessary for the repair of injuries, taking for illustration such wounds as are made in operations. Recent improvements in surgery have shown how right Hunter was in laying down the necessity of discriminating injuries—especially those which are exposed to contact with the air. He says:—"The injuries done to sound parts I shall divide into two sorts according to the effects of the accident. The first kind consists of those in which the injured parts do not communicate externally, as concussions of the whole body, or of particular parts, strains, bruises, and simple fractures, which form a large division. The second consists of those which have an external communication, comprehending wounds of all kinds, and compound fractures." And again, "The injuries of the first division, in which the parts do not communicate externally, seldom inflame; while those of the second commonly both inflame and suppurate." Here he has expounded the whole art of subcutaneous surgery. I might remind you of the difference of the processes of repair in simple and compound fracture; although the simple may be attended with less violence, yet from exposure to the air, if continued, they seemed to be far the worst; even after extreme violence the symptoms of suppuration are far less than in compound fractures. I found a number of examples of this truth in some experiments I made to see how the tendons were healed. In a certain number I made openings in the skin, and certainly the wound externally was more severe than in subcutaneous wounds. In a rabbit the tibialis anticus and the extensor longus digitorum were divided on the right side, with a wide section through the skin, and on the left with a subcutaneous section, through a small opening. Twelve days after it was killed; the subcutaneous wound had healed, and on the right side there was a large collection of pus on the wound, covered in by scabbing, and no trace of repair. The case is yet stronger, because in making a subcutaneous wound, the violence is greater than in making an open one. Still subcutaneous wounds seldom inflame; while open ones are much subject to this process and to suppuration. The reason for this difference is, that the materials for the repair of the two kinds of wounds are different. But before I speak of this I will just mention the different modes in which open injuries are healed:—1st. By immediate union. 2d. By primary adhesion. 3. By granulations. 4. By secondary adhesions. 5. By scabbing.

The process of immediate union corresponds with that of Hunter's "union by the first intention," and is accomplished by the effusion of coagulable lymph between the two old surfaces of the wound. Hunter's account of it was, that a bond of union was formed by the blood extravasated between the surfaces of the wounded parts: the blood organizes itself, and becoming vascular, forms a connecting tissue between them. Without doubt Hunter was in error in supposing that extravasated blood is used commonly for this purpose; but I will speak first of what share it really has. First, I may remind you of the evidences adduced in a former course for coming to the conclusion that blood may be organized. We may assume, that granules form a tissue and become vascular. I have already mentioned several cases in which organization in the tissue occurred—cases of clot forming in veins and becoming organized—cases of clots of blood in the heart organizing into tumors,—and clots above ligatures in tied arteries. Dr. Zwicky has clearly traced this process till it became part of the fibrous cord. A case of this kind occurred in the dura mater of an insane person examined by Mr. Holmes Coote,—beneath it there was a distinct layer of clotted blood like that which is effused in apoplexy of the cerebral membranes as described by Mr. Prescott Hewitt. A number of small vessels passed from the dura mater into this clot; and it was from these vessels, while filled with blood, that I made this sketch (diagram exhibited.) Here it bears a close resemblance to a sketch I have made of the vessels of an old adhesion; and the structure of the clot is interesting as being similar to the material used for adhesion. When acetic acid was added to it there appeared in it elongated corpuscles, and fibre with dark edges, bearing no disiinct granules. No such structures are found in the ordinary products of inflammation; but, from comparison, they bear an exact resemblance to the nuclei formed to develope ordinary cellular tissue. The examination of this specimen shows one stage of the clotted blood becoming vascular in the formation of the cellular tissue, and is of the same kind as that for the healing of subcutaneous injuries. With all this evidence that the blood becomes organized, it was with surprise that I found that blood effused and extravasated, can hardly ever become a bond of union in injuries. In a large majority of cases no blood is extravasated. Thus, in subcutaneous wounds of the tendons, it is very rare to find the blood extravasated. I find out of twenty cases in which I have divided the tendon Achillis in rabbits, only one in which a clot existed in the track of the wound. In subcutaneous division of the tendon the same thing holds, except where an artery is involved; and amongst fractures there is seldom so much blood effused as could form a bond of union. Here is the tibia of a man, whose legs were crushed by a railway carriage, in which there is no such quantity of blood as could form this bond. These cases, then, prove the fact that extravasated blood is not necessary as a bond of union. Still, when blood is extravasated, the question arises, what becomes of it. If the blood be enough to form a large clot, and the wound be exposed to the atmosphere, it is obvious that the blood will be cast out of the wound. But under favorable circumstances, the more ordinary mode is by absorption. But my observations have led me to dissent from the opinion generally entertained of the absorption of blood thus effused. The common idea is that the blood is absorbed and lymph takes its place. But this can hardly be, since the absorption of blood is a very slow process. In cases of fractures I have found the blood not absorbed in five weeks; and in a common leech-wound I have found the blood corpuscles all as perfect a month afterwards. The truth rather seems to be, that the blood is enclosed within the effused reparative material, and is by it finally absorbed. In one case the tendo Achillis was divided six days before the death of the animal. As repair was proceeding, on slipping through the length of the substance forming the bond of union, I found a perfect clot of blood, which had been effused from a large vessel, and the new material was formed completely round it, and absorbed it. This only confirms me in the opinion that the inclosure was formed of extravasated blood. In another case I found still within the reparative substance traces of blood corpuscles imbedded previous to its absorption. Still I should not be willing to believe that it would never be organized. Hunter states that he has seen extravasated blood forming a bond of union; and one does sometimes find blood so adhering to the bones that one can hardly believe it is about to be absorbed. The conclusion, then, as to the share blood takes in the repair of injuries, is this:—

1. That it is not essential or favorable to the healing of any wound.

2. That most commonly, if existing in large quantities, and exposed, it is ejected.

In favorable circumstances it may be absorbed, subsequent to the adhesion of the new material.

4. It may become organized and form part of the reparative material.

The material most commonly employed is that of coagulable lymph.

Our notions concerning the properties of this substance, when once effused for the repair of injuries, are almost entirely from examinations of the lymph effused in acute inflammations. The identity is far from being proved, but their similarity is in many particulars evident, and especially in that both manifest, by their spontaneous coagulation, that they contain fibrine. The coagulum, which is spontaneously formed in reparative material, is identical with that of fibrine : chemically, too, they appear the same. The natural tendency of coagulable lymph is to develope itself into common fibro-cellular tissue ; but in certain cases the developement of lymph passes beyond this form. Thus, for the repair of bone, the lymph may proceed a certain distance towards the developement of a fibrous tissue, or the lymph may proceed at once to the formation of a nearly perfect cartilage, and this may ossify. Where it is produced, it has characters adapted to the functions of the parts that it unites. The bond for the union of a tendon is much tougher than that for a common scar in the skin ; that in skin is tougher and less pliant that that in mucous membrane, and so on. It has been thought a nice question, whether these variations are determined by the properties of the lymph itself, or by an assimilating force in the parts in which it is deposited. I have little doubt it is such a germ-power as that which forms the embryo out of very different materials. We have yet no observations to show a difference in the characters of the materials effused for the repair of injuries of different parts, or in different circumstances ; and yet such a difference, in even the original characters of the lymph, is indicated by the fact, that it passes through two different ways of developement. The lymph, or new material, which is produced for the repair of open wounds, generally developes itself into fibro-cellular tissue through nucleated cells : that formed for the healing of subcutaneous wounds as generally developes itself into the same tissue through the medium of nucleated blastema.

It must not appear an objection, that there should be two modes of developement to the same perfect tissue ; for this is usual, and has been observed in nearly all the tissues. I spoke of it last year in relation to the developement of the blood corpuscles, of which a first set are formed from part of the embryo cells that form the germinal area or the whole body of the embryo, and the second set are formed exclusively from the corpuscles of lymph and chyle ; and so it is with the cartilage, the muscular and other tissues that are formed in the earliest periods of embryo-life. At first they are developed from some of the embryo cells ; yet in later life, no such cells are seen among them, but others appropriate to them, and of widely different form. So also in the bones, which at first are developed through cartilage, but in their subsequent growth are increased by ossification of fibrous tissue ; and in the repair of which we shall find even more numerous modifications of these different developements.

(Here several diagrams were exhibited, of embryo cells, exudation cells, and granulation cells.)

The cells first formed are round, very slightly granular, display a well-marked cell-wall, and a clearly-defined round nucleus. Whether in granulations, or in inflammatory exudations, such cells present a striking resemblance to the white corpuscle, or lymph corpuscles, in the blood. In the developement of cellular tissue from these cells, the first apparent change is in the nucleus. It becomes oval, even before the cell does, and at the same time becomes clearer and brighter. One or two nucleoli now appear distinctly in it, and soon it attenuates itself; but this it does later, or in a less degree, than the cell; for a common appearance is that of elongated cells bulged out at their middle by the nucleus. With these changes each cell also is developing its structure; first becoming more granular, then having its cell wall thinned, or even losing it. It elongates at one or both ends, and thus are produced a variety of lanceolate cells, which gradually elongate towards the filamentous form. As they thus change, they also group themselves, so that the swollen part of each, at which the nucleus lies, is engaged between the thinner parts of the two or more adjacent to it. The final disposal of these nuclei is hard to discern. In many instances, they are developed into filaments, like those of elastic tissue. But in the developement of the cellular tissue formed in inflammation or granulating wounds, the nuclei rather seem to waste, and be absorbed. Certainly such nucleus-fibres are not found in scars lately formed, though common in those of old standing. But, in their time, they display more organic activity than the cells,—for they take the initiative in the multiplication of the cells. In granulations, one may often find large compound cells, containing eight, ten, or more, nuclei. These have been derived by subdivision from the nucleus of the simple cell, and are probably destined to be the nuclei of as many separate cells. Such is the process for the developement of fibro-cellular tissue through nucleated cells, and though some modifications of it take place, yet none of them affect the essential characters of the process.

The developement of fibro-cellular or fibrous tissue through nucleated blastema is observed in the natural developement of tendons and ligaments. It is the same which Henlé regards as the only mode of developement of the fibro-cellular and fibrous tissues. Schwann, on the other hand, believes that there is no other mode than that which I have just described. Certain it is, that both these modes of developement of fibro-cellular or fibrous tissue may be traced; nor can any better example be selected than in the formation of scars from granulations, and of the bonds that unite subcutaneous divided tendons. Of the union of divided tendons I shall speak in a future lecture. For the present purpose, and in relation to the developement of fibro-cellular or fibrous tissue from nucleated blastema, it may be enough to state, that when the first effusion of the products of the inflammation, excited by the violence of the wound, is completed, then a quantity of finely molecular or dimly-shaded substance, like homogeneous or dotted fibrine, begins to appear in the space in which the bond of union is to be formed. This substance is infiltrated in the tissue that collapses into the space between the retracted ends of the tendon. At first there is no appearance of nuclei or cytoblasts in it; it seems to be merely a blastema of fibrine; but as it acquires firmness and distinctness, the nuclei appear in it; they seem to form out of collecting clusters of granules, and presently appear as oval bodies, with dark hard outlines, soon becoming elongated, with clear contents, without nuclei, irregularly scattered, but so firmly imbedded in the blastema, that, in general, they cannot be dislodged. Commonly, the application of acetic acid is necessary to make them distinct, by making the intermediate substance transparent, while the nuclei themselves acquire dark edges and shrivel up a little. The nuclei undergo little change, while the blastema in which they are imbedded, is acquiring, more and more distinctly, the filamentous appearance, and then the filamentous structure,—only they appear to elongate, and to attenuate themselves, and to grow more irregular in their outlines as if by shrivelling.

The blastema may become at length perfect fibro-cellular or fibrous tissue. The final disposal of the nuclei is doubtless sometimes that they are developed into the nucleus-fibres, and constitute some of the various forms in which elastic yellow tissue is found mingled with the proper white filaments. But, in the process of repair by tissue thus developed, as well as by that which is formed through cells, I believe that the nuclei finally shrivel.

The deduction from these facts appear to be, that the material of repair for subcutaneous wounds is developed into cellular tissue through the formation of nucleated blastema; while that for repair by primary adhesions, and by granulation, is developed into the same through nucleated cells. These instances, then, may enable us to conclude respecting the existence or non-existence of inflammation in the healing of injuries. The material of repair in union by primary adhesion and granulations is the same, in appearance and mode of developement, as we find produced in other conditions which are called inflammatory. But, in the healing of subcutaneous wounds, we find a different material of repair, which resembles, in both appearance and mode of developement that which we find forming growths whose production is not attended with any signs of inflammation. On these grounds, therefore, we are justified in holding, generally, that inflammation ensues in the healing by adhesion and by granulations, but does not exist in the healing of subcutaneous wounds. Although inflammation may be deemed necessary for the production of the lymph that may form adhesions or granulations, yet we haye no evidence that the continuance of inflammation is necessary for the organization of the lymph. Rather, the persistence of inflammation seems to hinder the organisation of the lymph, to keep it in a low state of developement, or to favor its degeneration—*Medical Times.*

